# Calcium silicate accelerates cutaneous wound healing with enhanced re-epithelialization through EGF/EGFR/ERK-mediated promotion of epidermal stem cell functions

**DOI:** 10.1093/burnst/tkab029

**Published:** 2021-09-30

**Authors:** Bingmin Li, Haowen Tang, Xiaowei Bian, Kui Ma, Jiang Chang, Xiaobing Fu, Cuiping Zhang

**Affiliations:** Research Center for Tissue Repair and Regeneration affiliated to the Medical Innovation Research Division and Fourth Medical Center of Chinese PLA General Hospital, 100048, Beijing, China; Department of Dermatology, Fourth Medical Center of Chinese PLA General Hospital, 100048, Beijing, China; Faculty of Hepato-Biliary-Pancreatic Surgery, Chinese PLA General Hospital, 100853, Beijing, China; Research Center for Tissue Repair and Regeneration affiliated to the Medical Innovation Research Division and Fourth Medical Center of Chinese PLA General Hospital, 100048, Beijing, China; Research Center for Tissue Repair and Regeneration affiliated to the Medical Innovation Research Division and Fourth Medical Center of Chinese PLA General Hospital, 100048, Beijing, China; Shanghai Institute of Ceramics, Chinese Academy of Sciences, 200050, Shanghai, China; Research Center for Tissue Repair and Regeneration affiliated to the Medical Innovation Research Division and Fourth Medical Center of Chinese PLA General Hospital, 100048, Beijing, China; Research Unit of Trauma Care, Tissue Repair and Regeneration, Chinese Academy of Medical Sciences, 100048, Beijing, China; Research Center for Tissue Repair and Regeneration affiliated to the Medical Innovation Research Division and Fourth Medical Center of Chinese PLA General Hospital, 100048, Beijing, China

**Keywords:** bioceramic, calcium silicate, epidermal stem cells, re-epithelialization, wounds

## Abstract

**Background:**

Human epidermal stem cells (hESCs) play an important role in re-epithelialization and thereby in facilitating wound healing, while an effective way to activate hESCs remains to be explored. Calcium silicate (CS) is a form of bioceramic that can alter cell behavior and promote tissue regeneration. Here, we have observed the effect of CS on hESCs and investigated its possible mechanism.

**Methods:**

Using a mouse full-thickness skin excision model, we explored the therapeutic effect of CS on wound healing and re-epithelialization. *In vitro*, hESCs were cultured with diluted CS ion extracts (CSIEs), and the proliferation, migration ability and stemness of hESCs were evaluated. The effects of CS on the epidermal growth factor (EGF), epidermal growth factor receptor (EGFR) and extracellular signal-related kinase (ERK) signaling pathway were also explored.

**Results:**

*In vivo*, CS accelerated wound healing and re-epithelialization. Immunohistochemistry demonstrated that CS upregulated cytokeratin 19 and integrin β1 expression, indicating that CS improved hESCs stemness. *In vitro* studies confirmed that CS improved the biological function of hESCs. And the possible mechanism could be due to the activation of the EGF/EGFR/ERK signaling pathway.

**Conclusion:**

CS can promote re-epithelialization and improve the biological functions of hESCs via activating the EGF/EGFR/ERK signaling pathway.

Highlights

- Calcium silicate is a biodegradable Si-containing bioceramic; it can accelerate cutaneous wound healing and facilitate re-epithelialization.
- *In vivo* study showed that calcium silicate increases the stemness of hESCs.
- Calcium silicate improved the proliferation and migration ability of hESCs and enhanced stemness of hESCs *in vitro*.
- The possible mechanism of calcium silicate exerts its role by upregulating the EGF/EGFR/ERK signaling pathway.

## Background

Skin is a protective organ that lies between the inner body and the external environment. As the outmost barrier, skin is vulnerable to various injuries [1]. Following wounding, skin initiates the healing process, which includes re-epithelialization, collagen deposition and angiogenesis [2]. The promotion of re-epithelialization helps restore the skin barrier function, thereby facilitating wound closure [3]. Therefore, improving the reconstructive strategies of epithelial tissues is of great value [4].

Human epidermal stem cells (hESCs), characterized by stemness and slow cycling, have been recognized as important reservoirs for epidermal regeneration during wound healing. Following injury, hESCs at the injured site are rapidly activated, subsequently initiating the re-epithelialization process [5]. Concurrently, neighboring hESCs are recruited to the wound site and participate in the process. Therefore, improving the biological functions of hESCs would benefit wound healing. However, direct stem cell therapy remains controversial [6]. It is of practical need to explore new methods that could alter stem cell behavior and thereby promote wound healing.

In tissue engineering, biomaterials have been widely proved to be able to alter the epigenetic status of cells and influence their behavior, which serves as a vital role in tissue repair, replacement and regeneration [7,8]. Although exact details remain to be studied, the extracellular signal-related kinase (ERK) has been proved to play an important role in biomaterial-induced differentiation of stem cells [9]. Katayama et al. demonstrated that enamel matrix derivative could induce mesenchymal stem cells (MSCs) osteoblastic differentiation and promote tissue regeneration via the upregulation of the ERK signaling pathway [10]. Moreover, the downregulation of the ERK signaling pathway in hESCs resulted in delayed wound healing [11]. Calcium silicate (CS) is a Ca-, P- and Si-containing bioceramic, which could facilitate tissue regeneration. Calcium silicate is biodegradable. After implantation, the Si ion could be detected in urine, blood, kidney, liver, and the biodegradation rate reached 97.17% after 26 weeks *in vivo* [12,13]. Yu et al. demonstrated that CS could promote angiogenesis by increasing the secretion of vascular endothelial growth factor (VEGF) and basic fibroblast growth factor (bFGF) [14]. Therefore, we reckoned that CS might be beneficial to skin wound healing. In addition, the impact of CS on hESCs has to date not been reported. Based on this, we hypothesized that CS exerts its promoting effect in re-epithelialization by the secretion of epidermis growth factor (EGF) as well as maintaining the stemness of epidermal cells.

In the present study, we explored the therapeutic effect of CS on wound healing and re-epithelialization *in vivo*. Subsequently, we investigated the effects of CS on hESCs behavior in terms of proliferation, migration and stemness. Moreover, the role of CS on the EGF and epidermal growth factor receptor (EGFR)-mediated ERK signaling pathway was also evaluated.

## Methods

### Mouse skin wound model and treatment with CS

The study protocol was approved by the Ethics Committees of PLA General Hospital. Forty 12-week-old female *Balb/c* mice were used in this study. The mice were anesthetized with 4% chloral hydrate before operation. After shaving, a 1 cm diameter full-thickness skin excision was performed on the back of each mouse. The mice were randomly divided into treatment group and control group. The treatment group were applied with CS-containing cream (the mixture of 0.015 g CS powder and 0.035 g artificial cell healing membrane) while the control group were smeared with 0.035 g artificial cell healing membrane. The cream was given at day 0, 3 and 5. At day 4, 7, 10, 14, five randomly chosen mice in each group were euthanized by cervical dislocation, the wounds were photographed and skin specimens were harvested. The wound closure rate was calculated as % of wound closure = 100 × (initial wound area − unhealed wound area)/initial wound area.

### The measurement of Si ion in wound tissue

The mouse skin tissue around the wound area was collected at day 7 after surgery. After being weighed, the sample was dissolved in 5 mL HNO_3_ and 1 mL HClO_4_. The solution was diluted with deionized water to 10 mL and the silicon content was measured with inductively coupled plasma mass spectrometry (ICP-MS).

### Histological analysis and immunohistochemistry staining

After fixing with 4% paraformaldehyde for 24 h, the collected specimens were dehydrated, embedded in paraffin and sectioned into 6 mm slices. The tissue sections were incubated with hematoxylin and eosin (H&E). Masson’s trichrome was stained to estimate the collagen deposition. Besides, the immunohistochemistry staining for cytokeratin 19 (CK19) and integrin beta-1 (ITGβ1) were used to evaluate the stemness of hESCs. All the slices were observed under the microscope.

### Cell culture and phenotype identification

The hESCs from human skin were purchased from Otwo Biotech Inc. (Shenzhen, China) and cultured in EpiLife medium (Gibco, M-EPI-500-CA) supplemented with 1% human keratinocyte growth supplement (HKGS) (S-001-5). Cell phenotype was detected by flow cytometry. Briefly, after the cells were fixed, permeabilized and blocked, they were incubated with CK19 and ITGβ1 for 30 min, followed by incubation with goat anti-mouse secondary antibody for 30 min. The fluorescence was detected by a flow cytometer. The antibodies including anti-cytokeratin 19 (CK19; ab7754), anti-integrin beta-1 (ITGβ1; ab24693) and goat anti-mouse IgG (H + L) (ab96879) were obtained from Abcam.

### Ion extracts preparation and ion concentration determination

The calcium silicate ion extracts (CSIEs) were prepared as previously described [15]. Briefly, the CS powder and the EpiLife medium were mixed in a 1:5 scale and incubated for 24 h. After centrifugation and filtration, the CSIEs were stored at 4°C for the downstream experiments. To determine the optimum dilution rate in hESCs, CSIEs were diluted with EpiLife media in the ratios of 1/32, 1/64, 1/128 and 1/256. The ion contraction of Ca, P and Si in each diluted product was detected by inductively coupled plasma-optical emission spectroscopy (ICP-OES).

### Cell viability measurement

The effect of CSIEs on cell viability was determined by a cell counting kit-8 (CCK-8) assay (Dojindo, Japan). Briefly, hESCs were seeded in 96-well plates and cultured in the diluted CSIEs products. One group that cultured in EpiLife medium was employed as control. After 1, 3, 5 and 7 days, 10 μL CCK-8 solution and 100 μL EpiLife medium were added to each well and incubated for 1 h. The absorbance at 450 nm of each well was measured with a microplate reader (BioTek, VT, USA).

### Cell cycle

A DNA content quantitation assay was employed to determine the cell cycle. The hESCs were cultured in the 1/64, 1/128, 1/256 CSIEs diluted products and the EpiLife medium for 72 h. Then, the cells were collected and fixed in 70% ethanol overnight. Subsequently, the cells were treated with 100 μL RNase A solution for 30 min and then mixed with 400 μL PI staining solution for 30 min. The fluorescence was detected with a flow cytometer and the percentage of cells in each phase was calculated.

### Cell proliferation assay

The EdU proliferation assay was carried out to detect the DNA synthesis. The cells were plated in 6-well plates and cultured in the specified medium as previously described. After incubation with 10 μM 5-ethynyl-2′-deoxyuridine (EdU) (BeyoClick EdU 594, Beyotime) for 2 h. Subsequently, the cells were fixed, permeabilized, washed and incubated with 0.5 mL click addictive solution for 30 min. Hoechst 33342 were stained to reveal the nuclei. The percentage of proliferative cells was by a fluorescence microscope. The EdU positive cells glow red while the nuclei glow blue under the microscope. The percentage of EdU positive cells was calculated.

### Cell migration assay

For the scratch assay, hESCs were seeded in the 6-well plates and cultured in EpiLife medium supplemented with 1% human keratinocyte growth supplement for 12 h. The culture medium was then changed to EpiLife medium without any supplement, and hESCs were cultured until reaching 90–100% confluence. Then, the monolayer in each well was scratched by a P200 pipette tip, washed with PBS and incubated with the specified medium. The pictures were taken at 0 h and 12 h to evaluate the migration area.

For the transwell assay, cells were suspended in EpiLife medium (without HKGS) and 100 μL cell suspension was added into the upper chamber of transwell 24-well plates (Corning) with a 8.0 μm polycarbonate membrane. Then 500 μL complete medium (containing 1% HKGS) supplemented with or without 1/64 CS CSIEs was added to the lower chamber. After 24 h, the cells that remained on the upper surface of the filter membranes were swabbed and those that had migrated to the lower surface were stained with 0.5% crystal violet for 10 min. The stained cells were observed under an optical microscope.

**Figure 1.**
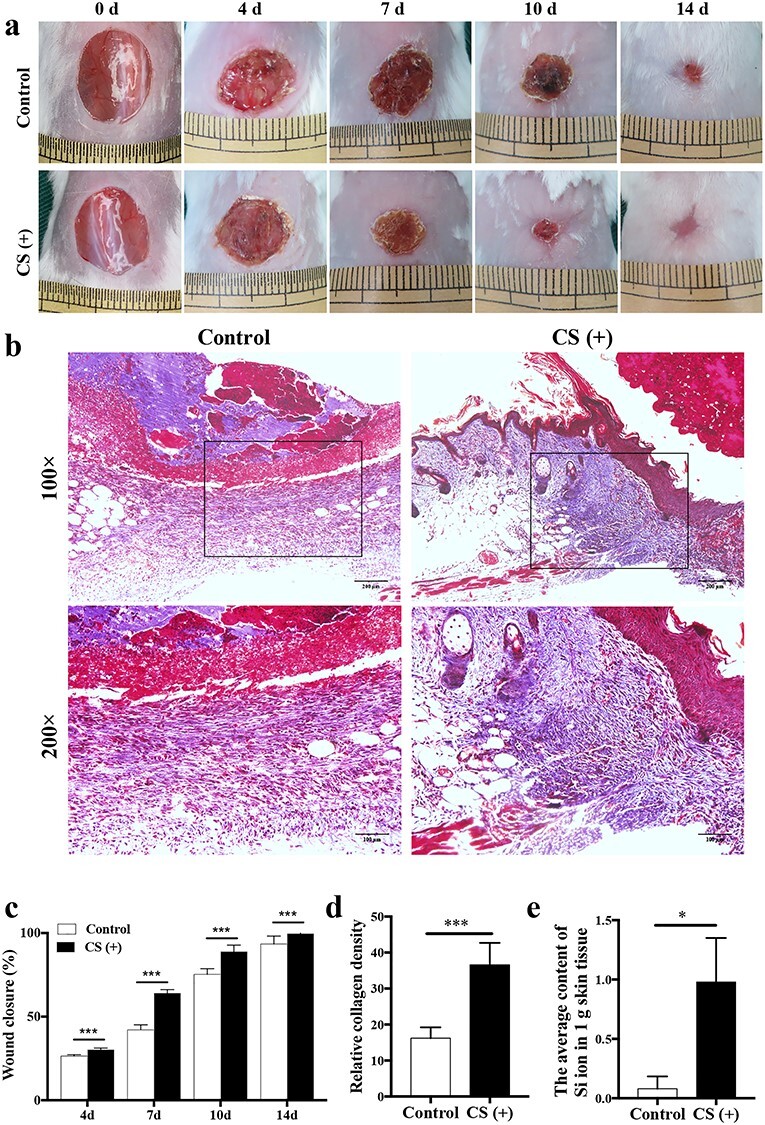
CS accelerates wound healing *in vivo*. (**a**) Healing progression of the wound from day 4 to day 14. (**b**) Masson’s trichrome staining of mice tissue at day 10 post-injury. The collagen fiber was stained in blue. Scale bar: 200 μm, 100 μm. (**c**) The statistical analysis of wound closure area in control and CS (+) group (n = 5). ^*^^*^^*^p < 0.001. (**d**) The statistical analysis of relative collagen density in control and CS (+) group (n = 5). ^*^^*^^*^p < 0.001. (**e**) The average content of Si ion in 1 g skin tissue in control and CS (+) group. ^*^p < 0.05. *CS* calcium silicate, *d* day, *Si* silicate

### Quantitative real-time polymerase chain reaction (qRT-PCR)

qRT-PCR was carried out to determine the difference in gene expressions. Briefly, cells were seeded to the 6-well plate and cultured by the specified medium for 3 days. Total RNA was extracted with TRIZOL reagent (invitrogen), cDNA was synthesized by FastKing gDNA Dispelling RT SuperMix (TIANGEN, China) and qRT-PCR was conducted by PreMix Plus SYBR Green (TIANGEN, China). Glyceraldehyde 3-phosphate dehydrogenase (GAPDH) was used as an internal standard. The primer sequences are as follows: EGF, forward, 5′-GTCTGCGTGGTGGTGCTTGTC-3′ and reverse, 5′- TGCGACTCCTCACATCTC TGCTC-3′; EGFR, forward, 5′- GTGTGCCACCTGTGCCATCC-3′ and reverse, 5′- GCCACCACCAGCAGCAAGAG-3′; KRAS, forward, 5′- AGTTGGAGCTGGTG GCGTAGG-3′ and reverse, 5′- TACTCCTCTTGACCTGCTGTGTCG-3′; Raf-1, forward, 5′- CTATGCGTCGTATGCGAGAGTCTG-3′ and reverse, 5′- GGTGC TGACCATGTGGACATTAGG-3′; ERK1, forward, 5′- CATTGTGCAGGACCT GATGGAGAC -3′ and reverse, 5′- GTTGGCGGAGTGGATGTACTTGAG -3′; GAPDH, forward, 5′- TATGACAACAGCCTCAAGAT −3′ and reverse, 5′- AGTCC TTCCACGATACCA −3′.

**Figure 2.**
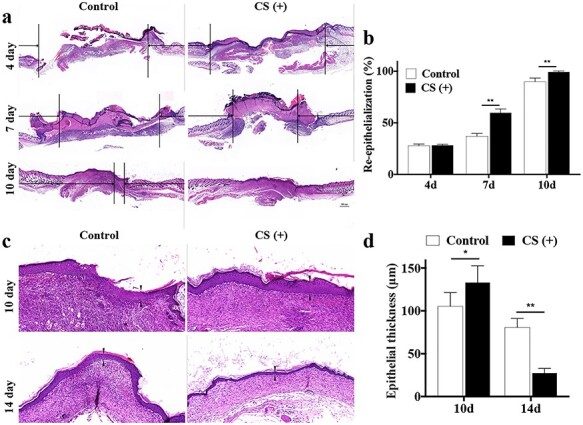
CS accelerates re-epithelialization. (**a**) H&E staining of mice tissue at day 4, 7 and 10 after surgery. Scale bar: 500 μm. (**b**) The statistical analysis of the epidermal gaps in each group (n = 5). ^*^^*^p < 0.01. (**c**) H&E staining of mice tissue at day 10 and 14 post-injury. The arrows indicate the thickness of the neo-epidermis. Scale bar = 500 μm. (**d**) The statistical analysis of the thickness of the neo-epidermis in the control and CS (+) groups (n = 5); ^*^p < 0.05; ^*^^*^p < 0.01. *CS* calcium silicate, *d* day, *H&E* hematoxylin—eosin staining

### Western blot analysis

Proteins were extracted by RIPA buffer (Invitrogen) and separated by 12% SDS-PAGE gels and transferred onto the Polyvinylidene Fluoride (PVDF) membrane. The blots were blocked with 5% non-fat dried milk, incubated with primary antibodies at 4°C overnight, and goat anti-mouse secondary antibody at 37°C for 2 h. The primary antibodies included anti-cytokeratin 19 (CK19; ab7754), Keratin 19 Mouse mAb (CST 4558 T), anti-integrin beta-1 (ITGβ1; ab24693) and anti-integrin beta-1 (ab179471). The bonds were developed by ECL kit (Solarbio) and exposure by the ImageQuant LAS 4000 system.

### Statistical analysis

All the data are shown as the mean ± SD. Statistical analysis was performed with SPSS Statistics (IBM, Armonk, NY). Two-way ANOVA was employed for comparison among groups at different time points. One-way ANOVA with Student–Newman–Keuls test was used for pairwise comparisons within a group, *p* < 0.05 was considered statistically significant. The result of the CCK-8 assay did not coincide with normal distribution. Therefore, a Kruskal-Wallis ANOVA was used for comparisons within groups.

## Results

### CS accelerates cutaneous wound healing in mice

A mouse full-thickness skin wound model was used to evaluate the effects of CS on wound healing *in vivo*. There was no significant difference in the wound area between the two groups at day 0. At day 7 and day 10, the mean wound area of the CS group was significantly smaller than that of the control group (*p* < 0.01). At day 14, all wounds in the CS group attained complete closure, whereas there remained detectable unhealed wound in the control group (Figure 1a, b). Masson’s staining demonstrated enhanced collagen deposition at day 10 (Figure 1c, d), with more collagen fibers in the wounds of CS-treated animals compared with those in the control group, which also confirmed accelerated wound-closure progress in the CS group. The Si ion in local wound tissue was also tested. At day 7, the average content of Si ion was 0.98 μg in 1 g skin tissue in the CS group compared with 0.23 μg in the control group (*p* < 0.05) (Figure 1e). These data indicate that CS can promote wound healing.

**Figure 3.**
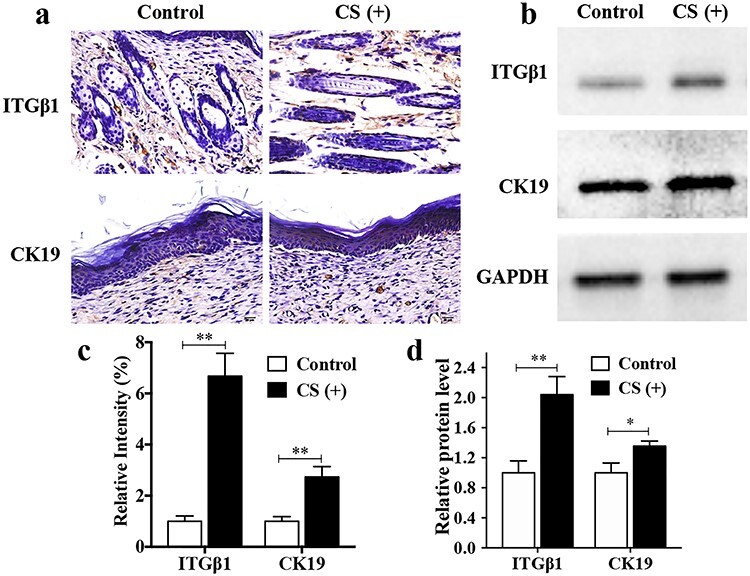
CS increases the stemness of hESCs *in vivo*. (**a**) Images of immunohistochemistry staining of CK19 and ITGβ1 at day 10 after surgery. CK19 positive cells were around the hair follicles and stained in brown. ITGβ1 positive cells were inside the epidermis and stained in brown. Scale bar: 20 μm. (**b**) The expression level of ITGβ1 and CK19. (**c**) The relative expression intensity of ITGβ1 and CK19 in control and CS (+) groups of the immunohistochemistry staining. ^*^^*^p < 0.01. (**d**) The relative protein level in control and CS (+) groups. The results were normalized to GAPDH expression. ^*^p < 0.05; ^*^^*^p < 0.01. *CK19* cytokeratin 19, *CS* calcium silicate, *GAPDH* glyceraldehyde 3-phosphate dehydrogenase, *hESCs* human epidermal stem cells, *ITGβ1* integrin beta-1

### CS accelerates re-epithelialization

The rate of re-epithelialization of both groups is presented in Figure 2. The epidermal gaps were smaller in the CS group at days 7 and 10 after wounding, which suggested a more rapid re-epithelialization rate (*p* < 0.01, Figure 2a, b). Regarding the thickness of neo-epidermis, at day 10, it was thicker in the CS group (133.2 ± 19.45 μm) than in the control group (105.4 ± 15.91 μm), indicating an accelerated re-epithelialization progression (*p* < 0.05). At day 14, the new epithelium of the CS group exhibited well-arranged and regularly distributed keratinocytes throughout the epithelium, which achieved the re-epithelialization. By contrast, the histological morphology of the neo-epidermis in the control group was irregularly arranged and hyperplastic (Figure 2c, d). These results accordingly confirmed that CS enhanced epithelial regeneration.

### CS increases the stemness of hESCs *in vivo*

Cellular stemness affects the biological functions of hESCs and thereby influences the re-epithelialization rate. CK19 is a specific marker for the bulge stem cell, which resides in the hair follicle. ITGβ1 is the marker for the interfollicular epidermis stem cells (HFSCs) population that is located in the basal layer of the epidermis [16,17]. During wound healing, the progenitors are rapidly activated and participate in the re-epithelialization process [5]. At day 10, CK19 staining around the hair follicles in the CS group was stronger than that in the control group (Figure 3a, c). Concurrently, ITGβ1 staining inside the epidermis was also stronger in the CS group than in the control group. The expression of ITGβ1 and CK19 in mouse skin tissue was also detected by Western blot analysis. As Figure 3b shows, ITGβ1 and CK19 expression were significantly enhanced in the CS group compared with the control group (*p* < 0.05). These data suggested that CS increased hESCs proliferation and stemness *in vivo*.

### Identification of hESCs and the effects of CSIEs dissolution products on hESCs proliferation

**Figure 4.**
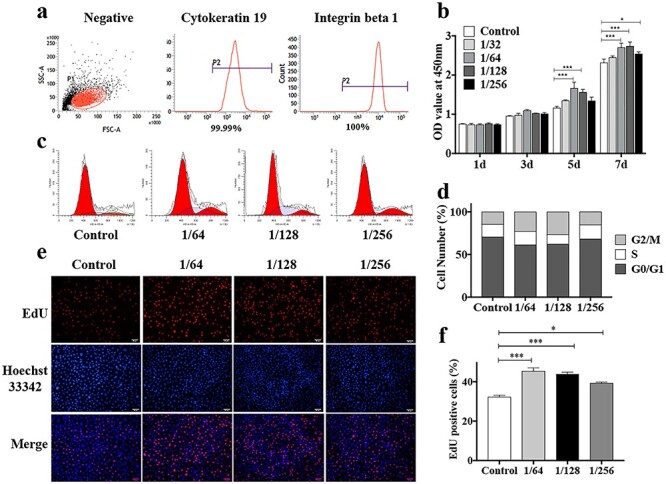
Identification of hESCs and the effects of CSIEs dissolution products on hESCs proliferation. (**a**) Flow cytometry demonstrated that the cells expressed a positive phenotype for CK19 and ITGβ1. (**b**) The statistical analysis of CCK-8 assay that reveals hESCs viability in four concentrations of CSIEs dilutions and EpiLife medium as a control (n = 5). ^*^p < 0.05, ^*^^*^p < 0.01, ^*^^*^^*^p < 0.001. (**c**) Flow cytometric analysis of cell cycle. (**d**) The percentage of hESCs in the G_0_/G_1_, S and G_2_/M phases, respectively (n = 5). (**e**) EdU positive cells (red) and nuclei (blue) in 1/64, 1/128, 1/256 CSIEs dilutions and EpiLife medium. Scale bar: 50 μm. (**f**) The statistical analysis of the percentage of EdU positive cells (n = 5). ^*^p < 0.05, ^*^^*^^*^p < 0.001. *CCK-8* cell counting kit-8, *CK19* cytokeratin 19, *CSIEs* calcium silicate ion extracts, *EdU* 5-ethynyl-2′-deoxyuridine, *G1* first gap, *G2* second gap, *hESCs* human epidermal stem cells, *ITGβ1* integrin beta-1, *M* mitosis, *OD* optical density, *S* synthesis

Flow cytometry revealed that the cells expressed a positive phenotype for CK19 and ITGβ1 (Figure 4a), which are specific molecular markers for hESCs [17]. The data showed that the cell morphology and phenotype described hESCs.

The CCK-8 assay was used to investigate the effects of CSIEs on hESCs viability and cytotoxicity. At days 5 and 7, the OD value of the 1/64 and 1/128 CSIEs dissolution products were higher than that in the other two groups (Figure 4b), showing that the 1/64 and 1/128 CSIEs dissolution products promote cell viability. To investigate the effect of CSIEs on cell cycles, the DNA content was determined by flow cytometry and the three cell subpopulations (G_0_/G_1_, S and G_2_/M) were calculated. A decreased G_0_/G_1_ subpopulation and accordingly increased S and G_2_/M subpopulations were shown in the 1/64 and 1/128 groups, indicating enhanced cell proliferative abilities in these two groups (Figure 4c, d). This result was further confirmed by EdU staining. The number of EdU-positive cells clearly increased in the 1/64 and 1/128 groups (Figure 4e, f). Furthermore, the Ca, P and Si ion concentrations in the diluted products were determined. The Si ion concentration differed among each group, which was 3.75 ± 0.05, 1.76 ± 0.02, 0.93 ± 0.02, 0.47 ± 0.01 and 0.05 ± 0.01 μg/mL in the 1/32, 1/64, 1/128, 1/256 group and control group, respectively (Table 1). It has been reported an optimal outcome will be obtained with an Si ion concentration range of 0.7 to 1.8 μg/mL [15]. Considering the results above, we used the 1/64 CSIEs dissolution products in the subsequent experiments.

**Table 1:** The ion concentrations of each dilution and EpiLife medium

	**Ca (μg/mL)**	**P (μg/mL)**	**Si (μg/mL)**
EpiLife medium	3.37 ± 0.26	44.93 ± 0.52	0.05 ± 0.01
1/32 CS	8.68 ± 0.32	42.73 ± 0.28	3.75 ± 0.05^*^
1/64 CS	6.02 ± 0.18	43.69 ± 0.49	1.76 ± 0.02^*^
1/128 CS	4.74 ± 0.29	44.10 ± 0.32	0.93 ± 0.02^*^
1/256 CS	4.04 ± 0.37	44.53 ± 0.21	0.47 ± 0.01^*^

^*^The difference in silicon ion concentration is statistically significant compared with control group (*p* < 0.05). *Ca* calcium, *CS* calcium silicate, *P* phosphorus, *Si* silicate

### CSIEs facilitate hESCs migration

**Figure 5.**
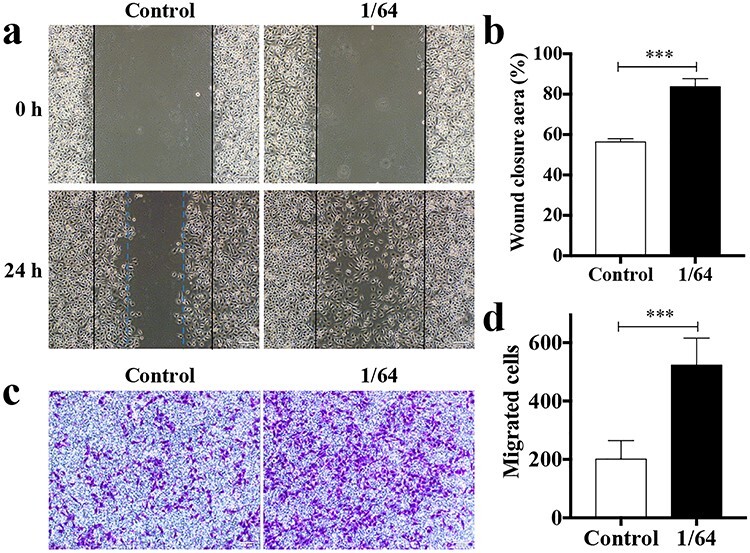
CSIEs facilitate hESCs migration. (**a**) 0 and 24 h after scratch. Scale bar: 100 μm. (**b**) The statistical analysis of wound closure aera (n = 5). ^*^^*^^*^p < 0.001. (**c**) Transwell assay. The photographs were taken at 24 h after cell seeding. Scale bar: 100 μm. (**d**) The statistical analysis of the number of migrated cells (n = 5). ^*^^*^^*^p < 0.001. *CSIEs* calcium silicate ion extracts, *h* hour, *hESCs* human epidermal stem cells

During wound healing, neighboring hESCs are recruited to the wound area and participate in re-epithelialization, therefore the migration ability is of vital importance. A scratch assay was performed to assess the effect of CSIEs on the migration of hESCs. The cellular migration area was significantly increased in the 1/64 CSIEs group compared with the control group, which demonstrated that CSIEs evidently enhanced hESCs motility (Figure 5a, b). The pro-migratory effect of CSIEs was further confirmed by the transwell assay. The number of migrated cells in the 1/64 CSIEs group was significantly increased compared with the control group (*p* < 0.01, Figure 5c, d). These results demonstrated that CSIEs facilitate hESCs migration.

### CSIEs enhanced stemness of hESCs *in vitro*

**Figure 6.**
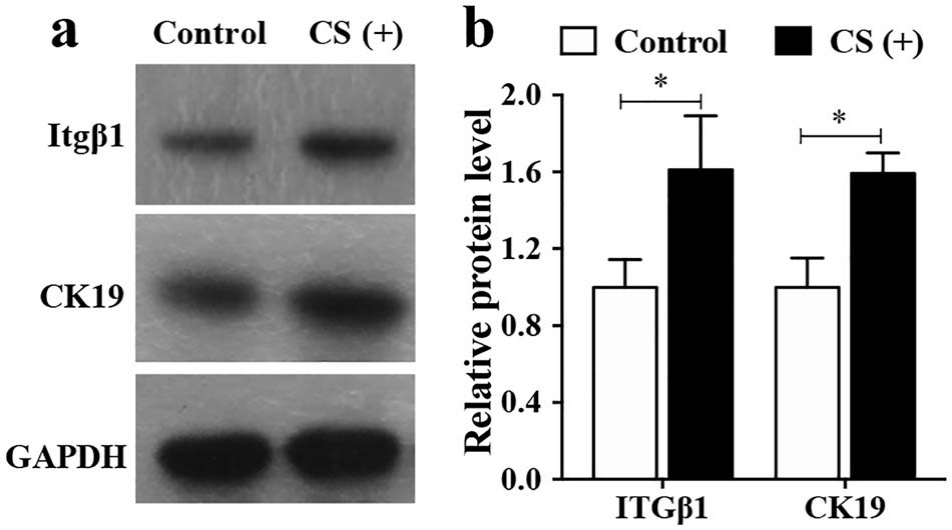
CSIEs enhanced stemness of hESCs *in vitro*. (**a**) The expression level of ITGβ1 and CK19. (**b**) The relative protein level in control and CS (+) groups. The results were normalized to GAPDH expression. ^*^p < 0.05. *CK19* cytokeratin 19, *CS* calcium silicate, *CSIEs* calcium silicate ion extracts, *GAPDH* glyceraldehyde 3-phosphate dehydrogenase, *hESCs* human epidermal stem cells, *ITGβ1* integrin beta-1

The stemness of hESCs determines the cellular biological functions, including proliferation and migration. To determine whether CSIEs can affect stemness of hESCs *in vivo*, ITGβ1 and CK19 expression was detected by Western blot analysis. ITGβ1 and CK19 expression was significantly enhanced in the CS group compared with that in the control group (*p* < 0.05) (Figure 6). The data suggested that CSIEs may exert their function by improving the stemness of hESCs.

### CSIEs exert their role by upregulating the EGF/EGFR/ERK signaling pathway

**Figure 7.**
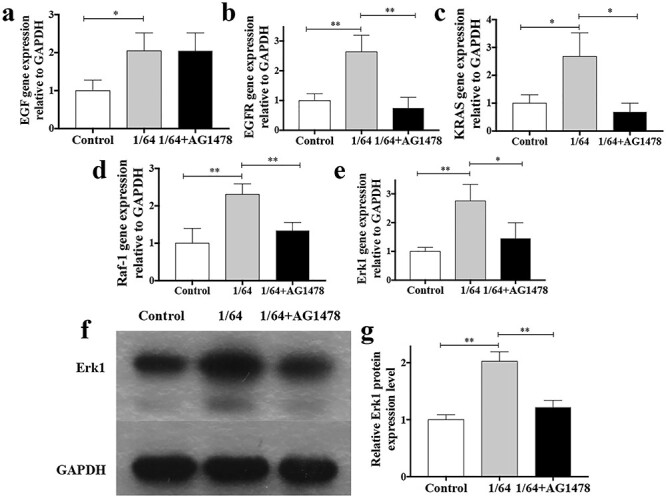
CSIEs exert their role by upregulating the EGF/EGFR/ERK signaling pathway. (**a**) The expression level of EGF assessed by RT-qPCR (n = 5). ^*^p < 0.05. (**b**) The expression level of EGFR assessed by RT-qPCR (n = 5). ^*^^*^p < 0.01. (**c**) The expression level of KRAS assessed by RT-qPCR (n = 5). ^*^p < 0.05. (**d**) The expression level of Raf-1 assessed by RT-qPCR (n = 5). ^*^^*^p < 0.01. (**e**) The expression level of ERK1 assessed by RT-qPCR (n = 5). ^*^p < 0.05, ^*^^*^p < 0.01. (**f**) The expression level of ERK1 in each group. (**g**) The relative protein level in each group. The result was normalized to GAPDH expression. ^*^^*^p < 0.01. *CSIEs* calcium silicate ion extracts, *EGF* epidermal growth factor, *EGFR* epidermal growth factor receptor, *ERK* extracellular signal-related kinase, *GAPDH* glyceraldehyde 3-phosphate dehydrogenase, *KRAS* one of the key human members, *Raf-1* C-Raf kinase, *Ras* protein subfamily, *RT-qPCR* quantitative reverse transcription polymerase chain reaction

Studies have shown that CS can induce the activation of growth factors, including bFGF, VEGF and EGF [14]. To detect the effect of CS on hESCs and their signaling pathways downstream, the expression of EGF and EGFR was detected. Furthermore, we investigated the effect on the ERK signaling pathway. The results indicated that CS upregulated EGF, EGFR, Ras, Raf-1 and ERK1 gene expression. While AG 1478, a specific EGFR inhibitor, antagonized the effect on EGFR, Ras, Raf-1 and Erk1. Erk1 protein expression in the test group also significantly increased compared with that in the untreated group (*p* < 0.05) (Figure 7). Therefore, activation of the EGF/EGFR/ERK signaling pathway may be the underlying mechanism in CS promoting hESC0s function.

## Discussion

In this study, we have reported that CS featured a therapeutic function in mouse skin wounds repair by accelerating re-epithelialization. Furthermore, we investigated the mechanism *in vitro* and found that (1) CS improved hESCs proliferation and migration ability; (2) CS upregulated the expression of CK19 and ITGβ1 in hESCs and therefore increased the cellular stemness; (3) the possible mechanism of CS facilitating wound healing may pertain to the upregulation of the EGF/EGFR/ERK signaling pathway. Our results suggest that CS improves hESCs biological functions and has a therapeutic effect on wounds.

During the wound healing process, delayed re-epithelialization inevitably results in higher incidence of infection and chronic wound formation. Therefore, accelerating wound closure is both a critical and difficult problem in the field of wound management [1,18]. In recent years, the application of biomaterials for tissue engineering has gained increasing attention. In particular, bioceramics have been reported to be of practical value in soft tissue wound repair [19,20]. The application of bioceramics on wounds also achieves promising outcomes. A recent study by Wang et al. revealed that akermanite, a kind of bioceramics, has a therapeutic effect on wounds [21]. The mechanisms of Si-containing biomaterials promoting wound repair may include (1) promotion of cell proliferation, (2) alleviation of inflammatory reaction, (3) stimulation of angiogenesis, and (4) anti-bacterial effects [4]. CS is a Ca-, P- and Si-containing bioceramic that can alter cellular behavior and promote tissue regeneration [22]. In this study, we have found that CS accelerates wound closure in mice. Histological analysis also confirmed a faster re-epithelialization rate. Therefore, we further investigated the mechanism of CS affecting re-epithelialization *in vitro*.

The participation of hESCs in the re-epithelialization process facilitated its proliferation and migration ability and thus would be beneficial to wound closure [23]. Our study showed that CS enhanced hESCs viability, drove more cells into the S and G2/M phases and thereby promoted cell proliferation. In addition, the scratch assay and transwell assay showed that CSIEs facilitated hESCs migration. These results suggest that CS can influence the cellular microenvironment and alter cellular behavior. In line with our results, previous works have reported that CS enhanced the proliferation and migration abilities of human umbilical vein endothelial cells (HUVECs) and fibroblasts [14,15]. The effector molecule is likely to be Si ion. CS achieves the optimum effect when the Si ion concentration ranges from 0.7 to 1.8 μg/ml [15].

It is recognized that stem cells are of vital importance in the treatment of wounds [1]. One important factor that influences the effect of stem cells on wound healing is cellular stemness [5]. Compared with earlier passages in cell culture, the aged hESCs have a higher threshold for Wnt signaling activation, which resulted in the delayed re-epithelialization [5]. Therefore, enhancing the stemness of hESCs is of benefit for wound healing [24]. CK19 and ITGβ1 are specific molecular markers of epidermal stem cells [16,24]. Previous research has demonstrated that increased expression of CK19 and ITGβ1 in hESCs showed a positive correlation with accelerated wound closure [16]. Further study confirmed that hESCs with heightened expression of CK19 and ITGβ1 increased colony-forming efficiency and exhibited better proliferative potential [24]. Moreover, ITGβ1 is crucial for epidermal cell migration, which is vital for re-epithelialization [25]. In our experiment, the expression of CK19 and ITGβ1 were upregulated both *in vivo* and *in vitro*, suggesting that CS improved cell stemness. In accordance with our finding, Wang et al. have reported that Aker, with a similar structure to CS, can also improve epidermal cell stemness by upregulating the Wnt/β-catenin pathway [21]. The Wnt/β-catenin pathway is crucial for the dedifferentiation process in epidermal cells [26]. In addition, Guan et al. reported that CS has an activation function on human urine-derived stem cells [27]. Therefore, we reckoned that the possible mechanism of CS promoting hESCs stemness may pertain to the upregulation of the Wnt/β-catenin pathway.

Previous studies have demonstrated that CS can stimulate the secretion of growth factors, such as VEGF, bFGF and EGF in HUVECs, fibroblast and hESCs, which can promote cell-to-cell communication and influence various cellular aspects, such as biological characteristics, gene expression and protein synthesis [14,28]. Moreover, there is a combined action between growth factors and integrins that can regulate cell adhesion, proliferation, differentiation and migration via the ERK signaling pathway [26]. Therefore, we examined the molecular markers of the EGF/EGFR/ERK signaling pathway. The result showed that the expressions of EGF, EGFR, Ras, Raf-1 and ERK were upregulated in the CS group, while the effect can be antagonized by AG1478, a specific EGFR inhibitor. In accordance with our finding, Wang et al. also showed that CS could upregulate the AMPK/ERK1/2 signaling pathway and promoted bone regeneration [9]. These data suggested that CS may exert its function by activating the EGF/EGFR/ERK signaling pathway. On the one hand, the ERK signaling pathway may help maintain the stemness in hESCs. The ERK signaling pathway has long been proposed as a critical pathway for cell dedifferentiation [24]. Grassian et al. reported that the upregulation of ITGβ1 in epidermal cells depends on Erk activity [29]. Moreover, Harrisingh et al. reported that Ras/Raf/ERK signaling can drive Schwann cell dedifferentiation [30]. On the other hand, the ERK signaling pathway plays an important role in accelerating wound healing. For example, Hu et al. reported that activation of ERK1/2 signaling enhanced the functional properties of fibroblasts and endothelial cells and thereby promoted cutaneous wound healing [31]. Moreover, the upregulation of the ERK signaling pathway can promote the migration ability of keratinocyte, and thereby facilitate re-epithelialization [2,32].

## Conclusions

In this study, we have demonstrated that CS can effectively promote wound healing and re-epithelialization in mice. Besides, CS improved the hESCs proliferation, migration ability and enhanced cellular stemness. The underlying mechanism might be the upregulation of the EGF/EGFR/ERK signaling pathway. Our findings suggest that CS may become a promising therapeutic tool for cutaneous wound healing, which could possibly be of practical use in the field of wound management.

## Authors’ contributions

BL and HT collected the data and wrote the manuscript; XB contributed to *in vivo* experimental tissue harvest; MS and KM contributed to the data analysis; CZ conceived the idea and designed the study; JC provided the experimental materials; XF contributed to the experiment design, financial support, and final approval of the manuscript. All authors read and approved the final manuscript.

## Abbreviations

bFGF: basic fibroblast growth factor; CCK-8: cell counting kit-8; CK19: cytokeratin 19; CS: calcium silicate; CSIEs: calcium silicate ion extracts; EdU: 5-ethynyl-2′-deoxyuridine; EGF: epidermal growth factor; EGFR: epidermal growth factor receptor; ERK: extracellular signal-related kinase; H&E: hematoxylin and eosin; hESCs: human epidermal stem cells; HKGS: human keratinocyte growth supplement; ICP-OES: inductively coupled plasma-optical emission spectroscopy; ITGβ1: integrin beta-1; MSCs: mesenchymal stem cells; qRT-PCR: quantitative real-time polymerase chain reaction; VEGF: vascular endothelial growth factor

## Funding

This study was supported in part by the National Nature Science Foundation of China (81830064, 81721092), the National Key Research Development Plan (2017YFC1103304), the CAMS Innovation Fund for Medical Sciences (CIFMS, 2019-I2M-5-059), the Military Medical Research and Development Projects (AWS17J005, 2019–126), and the Beijing Natural Science Foundation (7204309, 7202197).

## Ethics approval and consent to participate

All animal studies were conducted according to the guidelines of the Institutional Animal Care and Use Committee of the Chinese PLA General Hospital.

## Competing interests

The authors declare no conflicts of interest.

## References

[1] Gonzales KAU , Fuchs E. Skin and its regenerative powers: an alliance between stem cells and their niche. Dev Cell. 2017;43:387–401. 29161590 10.1016/j.devcel.2017.10.001 PMC5797699

[2] Ren S , Chen J, Duscher D, Liu Y, Guo G, Kang Y, et al. Microvesicles from human adipose stem cells promote wound healing by optimizing cellular functions via AKT and ERK signaling pathways. Stem Cell Res Ther. 2019;10:47. 30704535 10.1186/s13287-019-1152-x PMC6357421

[3] Plikus MV , Gay DL, Treffeisen E, Wang A, Supapannachart RJ, Cotsarelis G. Epithelial stem cells and implications for wound repair. Semin Cell Dev Biol. 2012;23:946–53. 23085626 10.1016/j.semcdb.2012.10.001 PMC3518754

[4] Kargozar S , Hamzehlou S, Baino F. Can bioactive glasses be useful to accelerate the healing of epithelial tissues? Korean J Couns Psychother. 2019;97:1009–20. 10.1016/j.msec.2019.01.028 30678892

[5] Li B , Hu W, Ma K, Zhang C, Fu X. Are hair follicle stem cells promising candidates for wound healing? Expert Opin Biol Ther. 2019;19:119–28. 30577700 10.1080/14712598.2019.1559290

[6] Han Y , Li X, Zhang Y, Han Y, Chang F, Ding J. Mesenchymal stem cells for regenerative medicine. Cell. 2019;8:886. 10.3390/cells8080886 PMC6721852 31412678

[7] Bratt-Leal AM , Carpenedo RL, Ungrin MD, Zandstra PW, McDevitt TC. Incorporation of biomaterials in multicellular aggregates modulates pluripotent stem cell differentiation. Biomaterials. 2011;32:48–56. 20864164 10.1016/j.biomaterials.2010.08.113 PMC2987521

[8] Yu Q , Chang J, Wu C. Silicate bioceramics: from soft tissue regeneration to tumor therapy. J Mater Chem B. 2019;7:5449–60. 31482927 10.1039/c9tb01467e

[9] Wang C , Lin K, Chang J, Sun J. Osteogenesis and angiogenesis induced by porous beta-CaSiO(3)/PDLGA composite scaffold via activation of AMPK/ERK1/2 and PI3K/Akt pathways. Biomaterials. 2013;34:64–77. 23069715 10.1016/j.biomaterials.2012.09.021

[10] Katayama N , Kato H, Taguchi Y, Tanaka A, Umeda M. The effects of synthetic oligopeptide derived from enamel matrix derivative on cell proliferation and osteoblastic differentiation of human mesenchymal stem cells. Int J Mol Sci. 2014;15:14026–43. 25123134 10.3390/ijms150814026 PMC4159837

[11] Zhang M , Zhang Y, Ding J, Li X, Zang C, Yin S, et al. The role of TrkA in the promoting wounding-healing effect of CD271 on epidermal stem cells. Arch Dermatol Res. 2018;310:737–50. 30209580 10.1007/s00403-018-1863-3

[12] Lin K , Liu Y, Huang H, Chen L, Wang Z, Chang J. Degradation and silicon excretion of the calcium silicate bioactive ceramics during bone regeneration using rabbit femur defect model. J Mater Sci Mater Med. 2015;26:197. 26099345 10.1007/s10856-015-5523-2

[13] Liu S , Jin F, Lin K, Lu J, Sun J, Chang J, et al. The effect of calcium silicate on in vitro physiochemical properties and in vivo osteogenesis, degradability and bioactivity of porous beta-tricalcium phosphate bioceramics. Biomed Mater. 2013;8:025008. 23428666 10.1088/1748-6041/8/2/025008

[14] Yu H , Peng J, Xu Y, Chang J, Li H. Bioglass activated skin tissue engineering constructs for wound healing. ACS Appl Mater Interfaces. 2016;8:703–15. 26684719 10.1021/acsami.5b09853

[15] Li H , Chang J. Bioactive silicate materials stimulate angiogenesis in fibroblast and endothelial cell co-culture system through paracrine effect. Acta Biomater. 2013;9:6981–91. 23416471 10.1016/j.actbio.2013.02.014

[16] Fu X , Sun X, Li X, Sheng Z. Dedifferentiation of epidermal cells to stem cells in vivo. Lancet. 2001;358:1067–8. 11589942 10.1016/S0140-6736(01)06202-X

[17] Dekoninck S , Blanpain C. Stem cell dynamics, migration and plasticity during wound healing. Nat Cell Biol. 2019;21:18–24. 30602767 10.1038/s41556-018-0237-6 PMC7615151

[18] Takeo M , Lee W, Ito M. Wound healing and skin regeneration. Cold Spring Harb Perspect Med. 2015;5:a023267. 25561722 10.1101/cshperspect.a023267 PMC4292081

[19] Yi M , Li H, Wang X, Yan J, Gao L, He Y, et al. Ion therapy: a novel strategy for acute myocardial infarction. Adv Sci (Weinh). 2019;6:1801260. 30643722 10.1002/advs.201801260 PMC6325593

[20] Kargozar S , Hamzehlou S, Baino F. Potential of bioactive glasses for cardiac and pulmonary tissue engineering. Materials (Basel). 2017;10:1429. 10.3390/ma10121429 PMC5744364 29244726

[21] Wang F , Wang X, Ma K, Zhang C, Chang J, Fu X. Akermanite bioceramic enhances wound healing with accelerated reepithelialization by promoting proliferation, migration, and stemness of epidermal cells. Wound Repair Regen. 2020;28:16–25. 31270882 10.1111/wrr.12742

[22] Li B , Bian X, Hu W, Wang X, Li Q, Wang F, et al. Regenerative and protective effects of calcium silicate on senescent fibroblasts induced by high glucose. Wound Repair Regen. 2020;28:315–25. 31943524 10.1111/wrr.12794

[23] Veltri A , Lang C, Lien WH. Concise review: Wnt signaling pathways in skin development and epidermal stem cells. Stem Cells. 2018;36:22–35. 29047191 10.1002/stem.2723

[24] Zhang C , Chen P, Fei Y, Liu B, Ma K, Fu X, et al. Wnt/beta-catenin signaling is critical for dedifferentiation of aged epidermal cells in vivo and in vitro. Aging Cell. 2012;11:14–23. 21967252 10.1111/j.1474-9726.2011.00753.x

[25] Grose R , Hutter C, Bloch W, Thorey I, Watt FM, Fassler R, et al. A crucial role of beta 1 integrins for keratinocyte migration in vitro and during cutaneous wound repair. Development. 2002;129:2303–15. 11959837 10.1242/dev.129.9.2303

[26] Moreno-Layseca P , Streuli CH. Signalling pathways linking integrins with cell cycle progression. Matrix Biol. 2014;34:144–53. 24184828 10.1016/j.matbio.2013.10.011

[27] Guan J , Zhang J, Guo S, Zhu H, Zhu Z, Li H, et al. Human urine-derived stem cells can be induced into osteogenic lineage by silicate bioceramics via activation of the Wnt/beta-catenin signaling pathway. Biomaterials. 2015;55:1–11. 25934447 10.1016/j.biomaterials.2015.03.029

[28] Kong L , Wu Z, Zhao H, Cui H, Shen J, Chang J, et al. Bioactive injectable hydrogels containing desferrioxamine and bioglass for diabetic wound healing. ACS Appl Mater Interfaces. 2018;10:30103–14. 30113159 10.1021/acsami.8b09191

[29] Grassian AR , Schafer ZT, Brugge JS. ErbB2 stabilizes epidermal growth factor receptor (EGFR) expression via Erk and Sprouty2 in extracellular matrix-detached cells. J Biol Chem. 2011;286:79–90. 20956544 10.1074/jbc.M110.169821 PMC3013038

[30] Harrisingh MC , Perez-Nadales E, Parkinson DB, Malcolm DS, Mudge AW, Lloyd AC. The Ras/Raf/ERK signalling pathway drives Schwann cell dedifferentiation. EMBO J. 2004;23:3061–71. 15241478 10.1038/sj.emboj.7600309 PMC514926

[31] Hu Y , Rao SS, Wang ZX, Cao J, Tan YJ, Luo J, et al. Exosomes from human umbilical cord blood accelerate cutaneous wound healing through miR-21-3p-mediated promotion of angiogenesis and fibroblast function. Theranostics. 2018;8:169–84. 29290800 10.7150/thno.21234 PMC5743467

[32] Pullar CE , Rizzo A, Isseroff RR. Beta-adrenergic receptor antagonists accelerate skin wound healing: evidence for a catecholamine synthesis network in the epidermis. J Biol Chem. 2006;281:21225–35. 16714291 10.1074/jbc.M601007200

